# microRNA and skeletal muscle function: novel potential roles in exercise, diseases, and aging

**DOI:** 10.3389/fphys.2014.00290

**Published:** 2014-07-31

**Authors:** John J. McCarthy

**Affiliations:** Department of Physiology, Center for Muscle Biology, University of KentuckyLexington, KY, USA

**Keywords:** microRNA, skeletal muscle, atrophy, hypertrophy, aging

Throughout the last decade, our understanding of the mechanisms involved in gene regulation has increased enormously, no more so than the role of non-coding RNAs (ncRNAs) in the regulation of gene expression (Cech and Steitz, 2014). In particular, a class of small ncRNAs, known as microRNAs (miRNAs), has emerged as having a central role in the post-transcriptional regulation of gene expression for a broad range of biological processes. The early discovery of muscle-specific miRNAs portended their importance in myogenesis, myopathies and adaptation to various forms of exercise (Lagos-Quintana et al., 2002). This timely Special Topic series summarizes the findings of studies investigating the function of miRNAs in skeletal muscle disease, aging and in response to exercise. Importantly, the series highlights important, unanswered questions in the field while also speculating on potential new functions for miRNAs in skeletal muscle biology.

Zacharewicz and colleagues provide a comprehensive review of studies that have described changes in miRNA expression following exercise, with different muscular disorders and aging (Zacharewicz et al., 2013). As clearly laid out in Table 1 of their review, numerous studies have reported changes in both muscle-specific miRNAs, known as myomiRs, and ubiquitously expressed miRNAs in response to both an acute bout of exercise, either resistance or endurance, and following training. A survey of Table 1 reveals, in general, that myomiR expression is increased following a bout of exercise whereas myomiR expression appears to be down-regulated with training, though exceptions to this pattern are shown. Although fewer studies have reported on the expression of non-myomiRs, these studies showed no consistent change in the miRNA expression pattern in response to either endurance or resistance exercise. In contrast to the findings from the exercise studies, miRNA expression profiling of various human myopathies identified a common set of miRNA that were up-regulated in each of the different muscular disorders. Subsequent studies found that myomiRs expression is altered in muscular dystrophy; myomiR-206 was shown to be dysregulated in both Duchene's muscular dystrophy and the *mdx* mouse model, consistent with the finding of Liu and coworkers that miR-206 knockout mouse shows an early onset of the dystrophic phenotype (Liu et al., 2012). Unfortunately, very few studies have examined the role of miRNAs in skeletal muscle aging but a study by Drummond and colleagues reported that let-7 miRNA expression was elevated in skeletal muscle of older humans (Drummond et al., 2010). The significance of this finding remains to be explored but given the anti-poliferative function of let-7 in the worm, the increase in let-7 expression may be a contributing factor to the alteration in satellite cell activity observed with age.

True to the early prediction that miRNAs would have a role in skeletal muscle, Sharma and coworkers provide a concise overview of myomiR function in myogenesis (Sharma et al., 2014). The canonical myomiRs miR-1 and miR-206 promote myoblast differentiation whereas, in contrast, miR-133a enhances myoblast differentiation. Importantly, how these miRNAs impact myogenesis is better understood than the aforementioned exercise studies because the target genes have been identified. In addition to myogenesis, the authors provide a list of miRNAs, and relevant target genes in Table 1 that have been reported to have a role in exercise, atrophy, aging and muscular dystrophy. The section covering miRNAs in muscle atrophy highlights the central role that miRNA have in regulating known pathways involved in atrophy. This is epitomized by the finding that the expression of the muscle-specific ubiquitin ligases Atrogin-1 and MuRF are regulated by miR-23a and, further, that myomiR-486 targets FoxO1 and PTEN, both upstream regulators of these known atrogenes. A review of the miRNA aging literature, offered evidence to suggest that miRNAs may have a role in maintaining the skeletal muscle phenotype and mass as well as limiting inflammatory cytokine production. Future studies will need to determine if the manipulation of miRNA expression is in fact able to ameliorate the loss of skeletal muscle function and mass known to occur with aging.

Hitachi and Tsuchida provide a focused discussion on miRNA regulation of signaling pathways involved skeletal muscle hypertrophy (Hitachi and Tsuchida, 2014). As nicely displayed in Figure 1 of their review, it is clear that myomiRs directly target components of the IGF-1/Akt/mTOR signaling pathway, the primary pathway known to regulate skeletal muscle hypertrophy. In particular, miR-1 has been shown to regulate expression of IGF-1 while miR-133 has been confirmed to target the IGF-1 receptor; consistent with these findings is the down-regulation of these myomiRs in response to a hypertrophic stimulus presumably contributing to enhanced IGF-1 signaling, thus helping to promote muscle growth (McCarthy and Esser, 2007). While shown to be involved in muscle atrophy, the muscle-specific miR-486 is proposed to have a role in muscle hypertrophy through targeting of PTEN, a well-established negative regulator of Akt activity. Based on work in the heart, the authors suggest the reasonable possibility that miR-208b and miR-499, members of the myomiR network as described by the Olson laboratory, have the potential to induce muscle hypertrophy through regulation of myostatin, a validated target gene (van Rooij et al., 2009). As pointed out by these authors, a better understanding of the exact role of miRNAs in the regulation of skeletal muscle mass will require the use of genetic models to disrupt miRNAs expression within the adult animal without affecting embryonic or post-natal development.

Nielsen et al. investigated the regulation of myomiRs in skeletal muscle aging (Nielsen et al., 2014). In human subjects, expression of miR-1 and 133a/b were found to be higher in older men compared to young men, and moreover, that miR-133a/b expression was further enhanced in old women. Based on these findings the authors tested the hypothesis that miR-133a/b expression is regulated by testosterone. In agreement with their hypothesis, evidence was presented showing that miR-133a/b expression was responsive to changes in testosterone levels in human and mouse skeletal muscle; however, additional data was presented showing that aerobic exercise appeared to independently regulate myomiR expression and that it was dominant to the effect of testosterone. As suggested by the authors, it would be interesting to know if the coordinated regulation of miR-133a/b by testosterone is direct via the androgen receptor or is through an alternative mechanism involving post-transcriptional processing of the primary miRNA transcript.

Historically, the study of miRNAs has rightly focused on their intracellular role in regulating gene expression; however, there is growing evidence that miRNA are present in the circulation and are capable of acting as a paracrine factor. Aoi and Sakuma review recent studies that have described circulating miRNAs (c-miRNAs) and how their levels change following exercise and under pathological conditions (Aoi and Sakuma, 2014). Although this area of research within the miRNA field is in its infancy, there is optimism that c-miRNA will be able to serve as biomarkers of athletic performance, physical fatigue and evidence of muscular diseases. To reach this lofty goal certain technical issues (standardized sampling and normalization) will have to be resolved as well as a better understanding of the mechanism of miRNA secretion and uptake by target cells. The studies on c-miRNAs, together with the studies reviewed in this Special Topics series, leave little doubt that miRNAs have not only an important role in skeletal muscle health and disease but also systemic health as well.

## Conflict of interest statement

The author declares that the research was conducted in the absence of any commercial or financial relationships that could be construed as a potential conflict of interest.
